# The Ebola Epidemic Crystallizes the Potential of Passive Antibody Therapy for Infectious Diseases

**DOI:** 10.1371/journal.ppat.1004717

**Published:** 2015-04-23

**Authors:** Arturo Casadevall, Liise-anne Pirofski

**Affiliations:** 1 Department of Molecular Microbiology and Immunology, Johns Hopkins Bloomberg School of Public Health, Baltimore, Maryland, United States of America; 2 Department of Microbiology and Immunology, Albert Einstein College of Medicine, New York, New York, United States of America; 3 Department of Medicine (Infectious Diseases), Albert Einstein College of Medicine, New York, New York, United States of America; Columbia University, UNITED STATES

The current Ebola epidemic provides a dramatic example of the potential of passive antibody therapy for infectious diseases that is also instructive of the hurdles and limitations involved in wide-scale reintroduction of this powerful anti-infective strategy. Passive antibody therapy was first used in the 1890s as "serum therapy" and was the first effective anti-infective therapy. Serum therapy was largely discontinued with the advent of antibiotic therapy in the early 1940s because it could not compete with regards to cost or ease of administration and had additional complexities, including that it had to be administered early in disease, it manifested lot-to-lot variation, and its efficacy required immune donors and the availability of a specific microbiological diagnosis so sera could be matched to the disease-causing microorganism [1]. Serum therapy using heterologous sera was also associated with "serum sickness," a syndrome caused by the formation of antigen-antibody complexes. However, antibiotic therapy was never shown to be superior in efficacy to antibody therapy and there were some conditions, such as pneumococcal pneumonia, where it may have had some advantages. Despite their wholesale abandonment, antibody therapies did retain a niche for certain conditions where no drugs were available, such as the prevention and/or treatment of tetanus, botulism, and certain viral diseases. The development of hybridoma technology and monoclonal antibodies (mAbs) in the mid-1970s promised to solve many of the problems of serum therapy, but, to date, there has not been formal reintroduction of antibody therapies for infectious diseases despite considerable and ongoing efforts to develop such therapies against viral diseases, such as HIV infection, and bacterial diseases, such as those caused by *Pseudomonas aeruginosa* and *Staphylacoccous aureus*. In contrast, mAbs have revolutionized the treatment of many cancers and rheumatic diseases and dozens have been licensed. Here we analyze why Ab-based therapies remain so underdeveloped for infectious diseases through the prism of the Ebola epidemic.

## The Ebola Epidemic of 2014

In 2014, an Ebola virus epidemic began in West Africa and it has affected over 17,000 individuals [2]. Unlike earlier Ebola outbreaks that were largely confined to isolated villages, this one struck in populated cities and was sustained through person-to-person transmission. At the end of 2014, the epidemic remained uncontrolled [2]. Currently, there are no drugs to treat Ebola, but, in the urgency and emergency triggered by the epidemic, two types of Ab-based therapies have been used: convalescent sera from patients who have recovered and a mAb cocktail known as ZMapp produced in plants [3]. Although at the time of this writing the news releases on the efficacy of Ab-based therapies have been largely favorable, the evidence is anecdotal and firm conclusions cannot be made until formal clinical trials are done, such as those encouraged by the World Health Organization [4]. Therefore, we will refrain from commenting on the efficacy of Ab therapies against Ebola virus and will focus, instead, on how this concurrent epidemic brings into focus the promise of this therapy while also highlighting the difficulties involved in reintroducing antibodies for the therapy of infectious diseases.

## Advantages and Limitations of Ab Therapies

Antibodies are molecules that can exert antimicrobial activity through different mechanisms that include promoting phagocytosis and Ab-dependent cellular cytotoxicity (ADCC), activating complement, neutralizing viruses and toxins, modulating inflammation, and affecting microbial metabolism (reviewed in [5]). This diversity of function makes it possible to tailor Ab therapies to specific diseases depending on what might be needed for protection. For example, antibodies overcome the anti-phagocytic properties of encapsulated microbes, such as *Streptococcus pneumoniae* and *Cryptococcus neoformans*, by promoting phagocytosis through Fc receptors while the toxin-neutralizing properties inhibit the deleterious effects of toxigenic bacteria, such as *Bacillus anthracis*. The versatility associated with Ab therapies is further amplified by the availability of different isotypes that differ in serum half-life and effector function, with the latter partially dependent on Fc receptors (FcR) that can amplify or reduce inflammatory responses. In this regard, the efficacy of broadly neutralizing antibodies to HIV is dependent on FcRs [6] and vaccine-elicited subclass selection, which could drive optimal FcR effector function of non-neutralizing antibodies against HIV [7]. Although several types of Ab therapies have been highly successful, it is noteworthy that, to date, available therapies have not taken full advantage of either Ab or FcR structural and functional diversity.

Historically, Ab preparations for therapy were obtained by immunizing animals or from recovered individuals as convalescent sera. Serum therapy for pneumococcus relied largely on horse immune serum. Animal sera were effective, but they could also elicit allergic reactions or the phenomenon of “serum sickness.” Human convalescent sera were used for viral diseases specific to humans, but such preparations were in short supply, and, in retrospect, carried the risk of inadvertent transmission of blood borne diseases. Nevertheless, in the latter half of the 20^th^ century, immune gamma globulin preparations were recommended in certain clinical settings for the prevention and therapy of diseases caused by Hepatis B virus, cytomegalovirus, rabies virus, tetanus toxin, and botulinum toxin, among others. Since the mid-1970s, monoclonal antibodies have been available and tools now exist for reducing the antigenicity of animal antibodies through humanization and for the generation of fully human Abs. Given that Abs are natural products, they have little inherent toxicity and the ability to reduce or eliminate their antigenicity means that side effects associated with serum therapy are significantly diminished.

Perhaps the greatest difference between Ab and conventional antimicrobial therapies is the exquisite specificity of most immunoglobulin molecules for their targets. Unlike most antimicrobial drugs that function indiscriminately against multiple species, antibodies target a single species and, often, a single serotype or variant within a species. This is a double-edged sword for the development of Ab therapies. Great specificity has the advantage that it targets only the offending microbe. For example, given that there is increasing evidence that antibiotic-induced disruptions of the microbiota are associated with deleterious effects in the treatment of bacterial diseases, the high specificity of Ab-based therapies offers a tremendous advantage. Regarding the limitation that highly specific Abs can fail to bind highly related variants, particularly for viruses, there are two strategies to overcome this problem: the development of Abs that target broadly neutralizing epitopes, as has now been demonstrated for HIV [8], dengue [9], and influenza viruses [10], and the generation of Ab cocktails composed of Abs against multiple serotypes or variants, as has been done for rabies virus [11] and *Clostridium difficile* toxin [12]. Similarly, although sera from patients who have recovered from Ebola virus disease can exhibit persistent neutralizing activity [13], antibodies against different Ebola virus strains often do not cross-react with other strains [14]. In the pre-antibiotic era, this was addressed by using serotype-specific sera that required isolating and typing the strain before instituting therapy. The problem is especially acute for mAbs, which recognize a single epitope, but this limitation can be bypassed by creating cocktails targeting various subtypes, although this increases the cost of research and development. MAb cocktails can also be designed to neutralize different targets with the goal of achieving higher efficacy through synergy. It is noteworthy that the experimental mAb treatment of Ebola, Zmapp, consists of a cocktail of three mouse–human chimeric Abs directed to the viral glycoprotein [3].

For reasons that are not fully understood, Ab therapies work best when given in a prophylactic mode (e.g., before infection) or early in the course of disease. For example, serum therapy for the treatment of pneumococcal pneumonia was effective only when given within the first three days of symptoms. In contrast, antimicrobial agents are often effective in established infection and disease. One proposed explanation for this limitation is that Abs work best in neutralizing the infective inoculum and cannot cope with the high microbial burdens of established infection [15]. An alternative explanation is that Abs work by altering the inflammatory response and once inflammation is established that it is difficult for Abs to exert their protective functions [16]. For viral diseases, the reduced efficacy of Abs in treatment mode could reflect a molar imbalance between Ab molecules and increasing numbers of viral particles, as well as the requirement for cell-mediated immunity to eradicate established infection. Whatever the explanation, the need for early administration is a limitation for therapy since this means a potential lack of efficacy in the setting of advanced disease. However, in contrast to antimicrobial therapy, which mediates protection only while the drug is pharmacologically available, the administration of Ab results in a state of immediate immunity that, combined with the long half-life of certain immunological molecules, can confer a long-standing state of reduced susceptibility.

In contrast to conventional antimicrobial therapies, Ab therapies can be developed extremely quickly and, sometimes, in the midst of an epidemic. For example, a potentially clinically useful mAb against the coronavirus responsible for the severe acute respiratory syndrome (SARS) was rapidly generated, in months [17], but was not used because the epidemic was contained. An even more expedient strategy is to use convalescent serum from survivors in an epidemic as a source of antibodies to treat those at risk and with concurrent disease. In the past, convalescent sera was used to treat influenza and Ebola virus disease [18,19]. Today, convalescent sera from survivors of Ebola virus disease has reportedly been used to treat cases, although details of how the sera have been used and evidence of their efficacy is anecdotal. Nonetheless, we note that there are at least three established mechanisms of antibody function that could benefit patients with Ebola: direct neutralization of Ebola virus, enhancement of Ebola virus uptake and/or killing by phagocytes, and modulation of Ebola-virus-induced inflammatory response. Regarding the latter, it is of interest that immunomodulation has been proposed as an intervention for Ebola virus [20] and anecdotal reports suggest that control of Ebola-virus-induced cytokine storm might be beneficial therapeutically.

## Underdevelopment of Ab Therapies for Infectious Diseases

Despite having pioneered the use of Ab therapy, the use of Ab-based therapies in the field of infectious diseases remains a work in progress despite enormous technological progress in Ab engineering and production. In contrast to oncology and rheumatology, where Ab therapies are now common, there are only two licensed mAbs for infectious diseases: one for the prevention of respiratory syncytial virus infection and the other for the therapy of anthrax. Hence, Ab-based therapies are severely underdeveloped for the treatment of infectious diseases. Although the causes for this are complex, we identify five factors that are, in some cases, interrelated and, yet, all work in synergy to hinder the widespread reintroduction of Ab therapies to this field.

- Cost. Ab therapies are generally significantly more expensive than small molecules because they must be produced in animals or cell culture. Immunoglobulins are proteins that require refrigeration for storage, which, in turn, increases their cost. However, when one considers the costs of antibiotic-associated colitis, damage to the microbiota, resistance in non-targeted organisms, and superinfections resulting from non-specific therapy, the cost accounting may be more favorable for Ab therapy.
- Specificity. As alluded to above, the specificity of Ab therapies means that they are pathogen specific and often target a subgroup of organisms within a pathogenic species. This limitation can be bypassed by creating mAb cocktails, but that, in turn, increases the complexity of production and cost. In addition, cocktails could face more complicated regulatory hurdles than therapies composed of a single active agent. Despite this, we note that mAb cocktails are being developed against Ebola virus, *C*. *difficile* colitis, and for the prevention of rabies.
- Market size and profitability. The antimicrobial spectrum of a drug combined with the prevalence of disease caused by the organisms for which it has efficacy determines its market size. This law of pharmaceutical economics has favored the development of broad-spectrum therapies that are responsible for widespread resistance and deleterious effects of Ab therapies on host microbiota. Hence, the exquisite specificity of mAb-based drugs de facto means smaller market sizes, which, in turn, increases costs, reduces profitability, and makes these reagents suitable for treating specific diseases unattractive to industry.
- The availability of existing therapies. For many infectious diseases, the current availability of effective therapy means that any attempt to develop Ab therapy involves establishing the advantage of such therapies either alone or in combination with existing therapies, and this greatly complicates clinical development and marketing. In other fields, where no therapy was available, the development of Ab therapies was easier because it provided something new. However, this situation may change since the declining efficacy of conventional antimicrobial therapies due to widespread resistance could create windows for the development of Ab-based therapies.
- Underdevelopment in diagnostics. The specificity of Ab therapies means that they will be effective only in situations where a precise microbial diagnosis is available. For bacterial diseases, the widespread availability of broad-spectrum antimicrobial drugs with relatively little toxicity created a culture of empiricism that has translated into underdevelopment in diagnostics. Consequently, microbial culture has remained the gold standard for the diagnosis of many infectious diseases for decades despite the availability of new technologies, such as nucleic acid amplification, that could have led to more rapid diagnosis. Fortunately, the situation is changing. An increasing recognition of the problems associated with broad-spectrum therapy combined with declining efficacy of such drugs due to widespread resistance is leading to the development and use of rapid diagnostic tools that could support the use of Ab-based therapies.

## The Near and Far Horizons

The ongoing Ebola epidemic provides a special lens for understanding the promise and roadblocks to the development of Ab-based therapies for infectious diseases, as well as ethical and cultural considerations that pertain to conducting clinical trials in the midst of an epidemic in under-resourced countries. Precedent for the use of convalescent sera during epidemics signals promise that trials of sera can be conducted even as the current Ebola epidemic rages [21]. We note that if a stockpile of effective Ab preparations against Ebola had been available early in the current outbreak, the administration of these preparations to contacts might have provided them with immediate immunity, possibly resulting in early containment of the epidemic and prevention of thousands of deaths. The fact that such preparations were not available in early 2014 reflects many factors, including cost, uncertainty about efficacy, lack of available markets, and the erroneous assessment, based on prior epidemics, that the threat would be small and localized. The suggestion has also been made that such therapies remain underdeveloped because they are unattractive to the pharmaceutical industry and to academic investigators, who may view them as not fitting with the current drug development model or not being cutting-edge science, respectively [21]. However, the fact that Ab-based therapies for Ebola are now in development, clinical trials of sera are being designed, and compassionate use of these therapies has been employed in the midst of the current emergency suggests that clinically useful preparations may be identified and available shortly.

For the near horizon, it is likely that Ab-based therapies will continue to make incremental advances in the repertoire of anti-infective strategies. Such areas include infectious diseases caused by drug-resistant organisms for which conventional therapies have lost efficacy and diseases for which there is no available therapy, such as Ebola. For example, the widespread use of broad-spectrum therapy has been associated with an increase in *C*. *difficile* colitis creating an opportunity for the development of toxin-neutralizing Ab therapy [22]. For the far horizon, we are optimistic that Ab-based therapies will be widely reintroduced as anti-infective agents given their inherent advantages in being natural products with low toxicity, high specificity, and established efficacy. Continued technological advances in the form of more efficient production strategies and alternative production sources, such as expression in plants, that could lower costs, combined with new therapeutic needs and better diagnostics, will make their use more attractive.
